# Thermal features, ambient temperature and hair coat lengths: Limitations of infrared imaging in pregnant primitive breed mares within a year

**DOI:** 10.1111/rda.13994

**Published:** 2021-08-16

**Authors:** Małgorzata Maśko, Olga Witkowska‐Piłaszewicz, Tomasz Jasiński, Małgorzata Domino

**Affiliations:** ^1^ Department of Animal Breeding Institute of Animal Science Warsaw University of Life Sciences (WULS – SGGW) Warsaw Poland; ^2^ Department of Large Animal Diseases and Clinic Institute of Veterinary Medicine Warsaw University of Life Sciences (WULS – SGGW) Warsaw Poland

**Keywords:** gestation, Konik Polski, seasonal fluctuations; hair coat length, thermography

## Abstract

Infrared thermography is a non‐invasive technique which allows to distinguish between pregnant and non‐pregnant animals. Detecting accurate body surface temperatures can be challenging due to external factors altering thermograph measurements. This study aimed to determine the associations between the ambient temperature, the hair coat features and the temperatures of mares' abdomens. It compared pregnant and non‐pregnant mares throughout 11 months. The research was carried out on 40 Konik Polski mares, which were divided into pregnant and non‐pregnant groups. The temperature (Tmax, maximal; Taver, average; Tmin, minimal) of the mares' abdomen was evaluated in two regions of interest: the whole area of the lateral surface of the mares' abdomen (Px1) and the flank area of the lateral surface of mares' abdomen (Px2). During the increasing period, the slopes in the linear regression equation did not differ significantly for ambient (Tamb) and surface temperatures in both groups. In the decreasing period, the slopes did not differ significantly for Tamb and Tmax in the non‐pregnant group. They also did not differ for Tamb and Taver in Px1 and Tamb and Tmin in Px1 in both pregnant and non‐pregnant groups respectively. Other slopes varied significantly (*p* < .001). There was no evidence of parallel changes in hair coat features and measured temperatures. The flank area appears more suitable for thermal imaging in pregnant mares due to the seasonal fluctuations in hair coat lengths.

## 1 INTRODUCTION

The diagnosis of pregnancy in domestic livestock is mostly based on rectal palpation and ultrasound rectal examination (Bucca et al., 2005; McCue, 2014). Foetal electrocardiography, ultrasonography and Doppler ultrasonography represent the available advanced technologies that enable a biophysical assessment of an equine pregnancy (Bucca et al., 2005). When the rectal or physical examination is difficult or impossible to perform, biomarkers in the blood may be used to determine the pregnancy and assess the risk of embryonic death or abortion (McCue, 2021; Shikichi et al., 2017). However, all the above techniques require direct contact with the animal, involve complex procedures or have invasive sample collection. Therefore, an indirect approach of studying pregnancy in wildlife or other captive equids is still required (Hilsberg et al., 1997).

Several studies have documented the progress of pregnancy in wild equids by analysing urinary (Monfort et al., 1991; Schook et al., 2013) or faecal reproductive hormone concentrations (Asa et al., 2001; Skolimowska et al., 2004; Ncube et al., 2011; Kozlowski et al., 2018). Although urine and faecal samples are efficient at assessing the reproductive status in captive equids, there is limited use of these techniques when investigating free‐range animals (Schwarzenberger et al., 1996). Therefore, a non‐invasive collection of faecal samples has been deemed the better choice for estimating the quantity of progestagens and oestrogens in equids (Schwarzenberger et al., 1996; Kozlowski et al., 2018). Other studies have used infrared thermography as a non‐invasive technique to distinguish between pregnant and non‐pregnant animals by imaging surface temperature gradients from the abdomen (Durrant et al., 2006; Jones et al., 2005; Bowers et al., 2009). It has been suggested that internal factors such as alterations in regional blood flow, the proliferation of tissues, metabolic and/or hormonal interactions associated with pregnancy cause differences in temperatures between pregnant and non‐pregnant animals which can be registered with infrared thermography (Bowers et al., 2009; Hilsberg et al., 1997). Therefore, thermography has been broadly used to recognize pregnancy in captive animals, for example, dairy heifers (Jones et al., 2005) or mares (Bowers et al., 2009), as well as in wildlife animals, for example, Grevy zebras, black rhinoceros (Hilsberg et al., 1997), giraffes (Hilsberg et al., 2002) and giant pandas (Durrant et al., 2006). Nonetheless, there are many limitations of non‐invasive imaging in the assessment of pregnant mares. There are internal and external factors acting simultaneously which can alter thermograph measurements. The external factors, which include the fluctuations in ambient temperature (Satchell et al., 2015; Soroko et al., 2017), sunlight exposure, air movement (Schutz et al., 2011; Soroko & Howell, 2018), debris on the imaged body surface (Montanholi et al., 2015) and thermal properties of the animals' hair coat (Domino et al., 2020; Jørgensen et al., 2020) may mask the underlying biological target by causing unexpected changes in the pattern of surface temperature.

Recent research about infrared thermography in pregnant equids has not considered the changes that occur on the hair coat. Therefore, this study sought to understand the relationship between temperature parameters and hair coat lengths. An animal's total insulation involves muscle, fat, skin and the hair coat (Cymbaluk, 1994). The latter is the most variable and is highly dependent on the ambient temperature. In most of the horses, the longest winter coats (Jørgensen et al., 2020) coincide with late pregnancy (Fowden et al., 2020), which is also the time when thermography has been proven the most useful (Bowers et al., 2009). Therefore, we hypothesized that body surface temperatures are related to the internal conditions of the animal, such as an increased metabolism (Hodgson et al., 1993; Witkowska‐Piłaszewicz et al., 2020) or changes in blood flow during pregnancy (Bowers et al., 2009; Winsor, 1971). We also hypothesized that body surface temperatures affect the thermal properties of the skin and hair coat (Domino et al., 2020) and the thermal gradient between the skin surface and the environment (Satchell et al., 2015; Soroko et al., 2017). Thus, this study aimed to understand the associations between ambient temperatures, hair coat features and the temperatures of the lateral surface of the mares' abdomens, by comparing pregnant and non‐pregnant mares throughout 11 months.

## 2 MATERIALS AND METHODS

### 2.1 Animals

The research was carried out on 40 Konik Polski mares. Mares were selected from a herd of 90 Konik Polski horses at the Polish state stud farm Dobrzyniewo. There were two distinct groups of mares: pregnant and non‐pregnant. The pregnant group was composed of 26 non‐lactating mares (*n* = 26; age 6.28 ± 4.04 years; height 142.40 ± 2.12 cm) whereas the non‐pregnant group contained 14 non‐lactating mares (*n* = 14; age 5.47 ± 3.90 years; height 143.10 ± 2.09 cm). All mares represented the same light level of hair coat colour (Mousy‐grey). The pregnant group's inclusion criteria were mares that had naturally mated in February and/or March and had a confirmed ultrasonographical pregnancy screened at 14‐ and 35‐days post‐ovulation, according to McCue's (2014) protocol. A detailed reproductive tract examination was conducted using an ultrasound scanner (MyLabOne; ESAOTE, Italy) and a linear 5 MHz transducer (ESAOTE, Italy). The non‐pregnant group's inclusion criteria were mares that did not mate during the current reproductive season and had who had an ultrasonographical exclusion of pregnancy. Before examining the reproductive tract, each mare had a basic physical examination to exclude any clinical symptoms of disease. This basic clinical examination measured rectal temperature, heart rate, respiratory rate and capillary refill time. It also evaluated the mucous membranes and lymph nodes. Only healthy mares were included into both study groups. Results on thermal parameters were previously documented (Maśko et al., 2021). This protocol was approved by the II Local Ethical Committee on Animal Testing in Warsaw on behalf of the National Ethical Committees on Animal Testing (No WAW2/007/2020, day 15.01.2020).

At the stud farm in Dobrzyniewo, all the studied horses were housed under the same conditions in all‐day open stables. They were fed twice a day with a personal dose of hay to maintain a healthy condition and had above 12h of daily access to a large grassy pasture. Throughout pregnancy, thermographic images, ambient temperature (Tamb), humidity and hair coat samples were measured monthly. Data collection began in February and was conducted until the last foaling took place in January.

### 2.2 Thermal imaging

The infrared thermal images of the lateral surface of the mares' abdomens were conducted using a non‐contact thermal camera (FLIR Therma CAM E60, FLIR Systems Brasil, Brazil) with a 0.99 emissivity and a temperature range from 10.0 to 40.0℃. To minimize the impact of external conditions (Satchell et al., 2015), the imaging was performed in a closed space, devoid of wind and sun radiation. The same researcher took a total of 880 images on the right and left side of the mare's abdomen after standard imaging area preparation (which consisted of brushing off dirt and mud 15 min before imaging) (Soroko et al., 2017). The placement of the thermal camera was always set at the same distance (2.0 m between the camera and the imaged area) and position (half of the vertical line through the tuber coxae).

**FIGURE 1:**
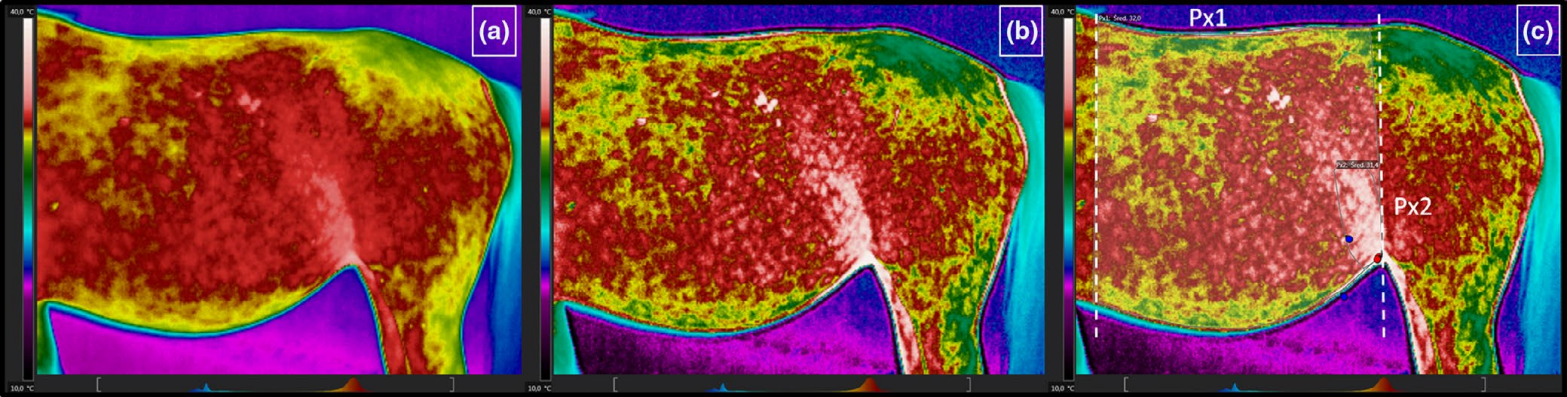
Protocol of thermal images analysis. The raw thermal images (a), thermal image after digital enhancement of details (b), thermal image with annotated the whole area of the lateral surface of the mares' abdomen (Px1) and the flank area of the lateral surface of mares' abdomen (Px2)

The temperatures of the lateral surface of the mares' abdomen were evaluated in two regions of interest: the first measurement took the whole area of the lateral surface of the mares' abdomen into account (Px1), whereas the second recorded the temperature of the flank area of the lateral surface of the mares' abdomen (Px2) (Figure 1). The raw thermal images (Figure 1a) underwent a correction by using the digital enhancement of details function (Figure 1b). The Px1 was annotated using a polygon measurement tool which considered the area from the vertical line lying behind the withers to the vertical line behind the tuber coxae. The top part of the Px1 region was bound by the edge of the back and the bottom edge of the abdomen. The Px2 region was annotated by magic tool measurement in the flank area. The limits of the Px2 region were the vertical line behind the tuber coxae and the edge of the last rib. The Px2 area had the highest temperature with a set tolerance threshold of 0.3℃. The range of the Px2 was adjusted to each individual mares’ thermal pattern (Figure 1c). Five thermal features were recorded: the maximal temperature (Tmax) in Px1 and Px2, the average temperature (Taver) in Px1, the average temperature (Taver) in Px2, the minimal temperature (Tmin) in Px1 and the minimal temperature (Tmin) in Px2. The maximum temperature parameter was combined due to the equal measurements in Px1 and Px2. All thermal measurements were calculated using the professional software SENSE Batch (SENSE Software, Poland).

### 2.3 Hair coat sampling and analysis

The hair coat samples were taken from the mid‐neck, approximately 5 cm below the base of the mane, by following the protocol described by Osthaus et al. (2018). The collected hair coat samples included the roots and were subsequently placed into individual tubes. The individual hair length was determined from a random sample of 10 pulled strands.

### 2.4 Statistical analyses

Data on thermal and hair coat length was presented in the form of data series. The hair coat index (HC Index) was calculated as the difference between the highest value of the hair length in the group (6.5 cm) and the individual hair lengths. Each month, data series were tested independently for univariate distributions using a Shapiro–Wilk normality test. Data comparisons showing normal distributions were assessed by a Repeated measures one‐way ANOVA with Geisser–Greenhouse correction, then by Tukey's multiple comparisons test or unpaired *t*‐test with Welch's correction. The non‐Gaussian data were evaluated by the Friedman test, which was followed by Dunn's multiple comparisons test or the Mann–Whitney test. To compare the data series as paired data between months of examination, the repeated‐measures one‐way ANOVA and the Friedman test were performed. The unpaired *t*‐test with Welch's correction and the Mann–Whitney test were used to determine whether there were differences between the pregnant and non‐pregnant groups. All the numerical data, except Tamb and humidity, was presented on plots as mean ± *SD*.

To calculate linear regressions, all 11 repetitions of the data series were divided into an increasing period (from the 1st to the 6th month) or a decreasing period (from the 7th to 11th month). Linear regressions were calculated for the Tamb, HC Index and each thermal feature (Tmax, Taver in Px1, Taver in Px2, Tmin in Px1 and Tmin in Px2). There were three regression equations for given data pairs (Tamb and each temperature; Tamb and HC Index; HC Index and each temperature) presented on each plot. All the slopes were significantly non‐zero (*p* < .001). The slopes within data pairs were also compared. If the difference between slopes was not significant (*p* > .05), one single slope measurement was calculated for all the data, and then the intercepts within data pairs were compared. When differences between the intercepts were not significant (*p* > .05), one intercept for all the data was calculated. The statistical analyses were performed using GraphPad Prism6 software (GraphPad Software Inc., United States). The significance level was established as *p* < .05.

Tmax, Taver and Tmin in Px2 were then used to determine threshold values and calculate the sensitivity and specificity for thermographic pregnancy diagnosis. To determinate temperature threshold, six temperatures were used beginning from the maximal value of Tmax, Taver and Tmin in Px2 by decreasing the value by 0.5℃. Temperature thresholds were set for month 6 to 11 independently for each feature in Px2. The mare was annotated as pregnant (1) when the individual temperature was above threshold, and annotated as non‐pregnant (0) below it. The same annotation was done in pregnant and non‐pregnant groups. Then the sensitivity (Se), specificity (Sp), positive predictive value (PPV) and negative predictive value (NPV) of thermographic pregnancy diagnosis were estimated. The values of Se, Sp, PPV and NPV were calculated across the range of pregnancy proportions from 0.1 to 1.0 using standard formulae (Dohoo et al., 2009).

## 3 RESULTS

**FIGURE 2:**
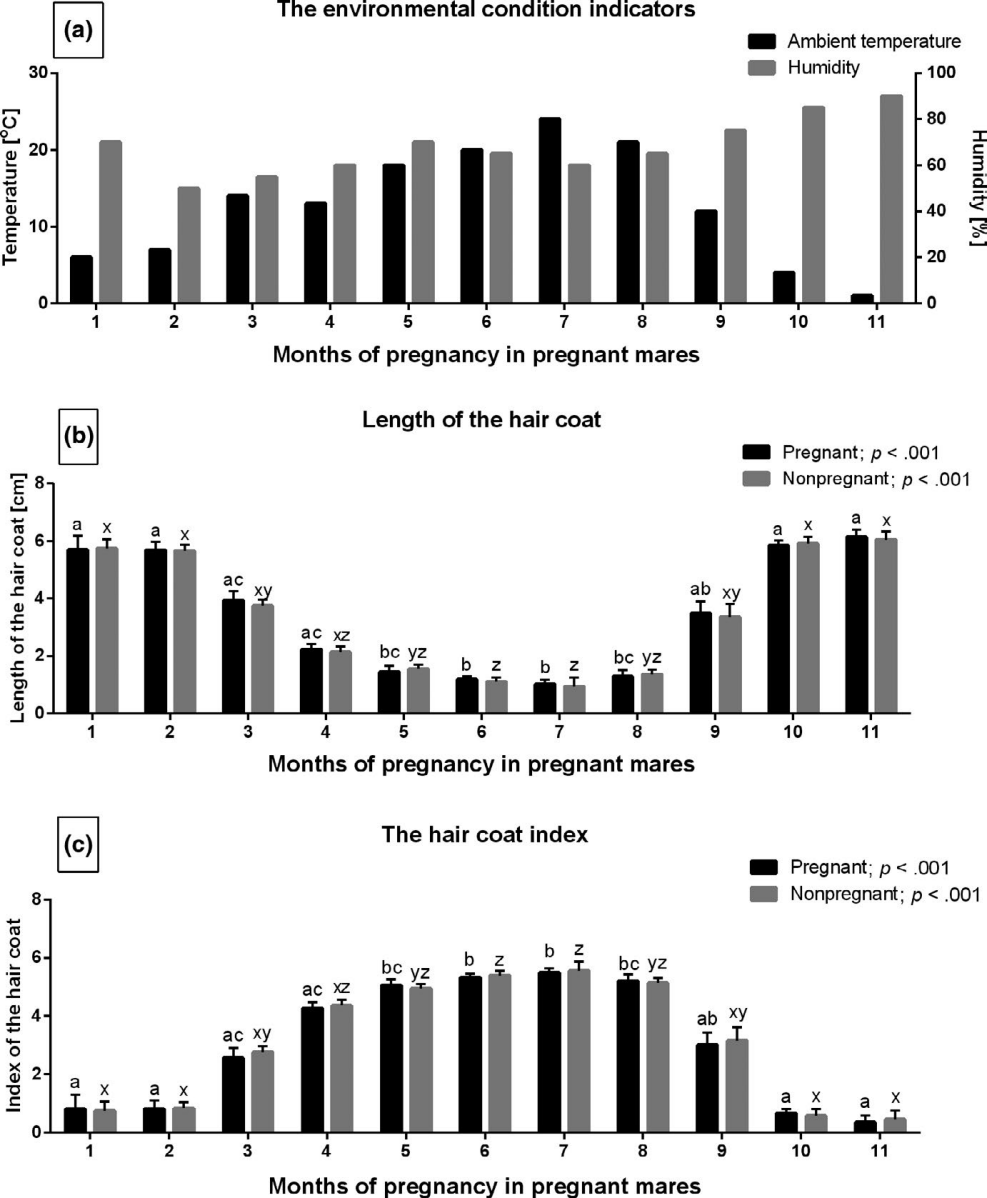
The environmental condition indicators (Tamb; humidity) (a), the hair coat length (b) and the hair coat Index (c) in consecutive months of study in pregnant (*n* = 26) and non‐pregnant (*n* = 14) groups. Bars represent values (a) or mean ± *SD* (b, c). Different superscripts within pregnant (a, b, c) and non‐pregnant (x, y, z) groups were statistically different (*p* < .05)

During the 11 consecutive study months, the Tamb ranged from 1.0℃ to 24.0℃. The reported values for the humidity index were between 50% and 90% (Figure 2a). Meanwhile, the length of the hair coat (Figure 2b) and the HC Index (Figure 2c) differed significantly (*p* < .001). However, hair coat features did not differ between pregnant and non‐pregnant groups, independently of the studied month. The longest hair coats occurred during the first two and the last two months which coincided with the highest HC Index values. The length of the hair coat decreased gradually from the 3rd to the 6th month and then increased from the 7th to the 10th study month. The hair coat was shortest during the 5th and 8th months. The changes in the HC Index followed the exact patterns of change as the length of the hair coat. The lowest values were recorded during the 1st, 2nd, 10th and 11th months, whereas the highest reported values occurred during months 5 and 8.

**FIGURE 3:**
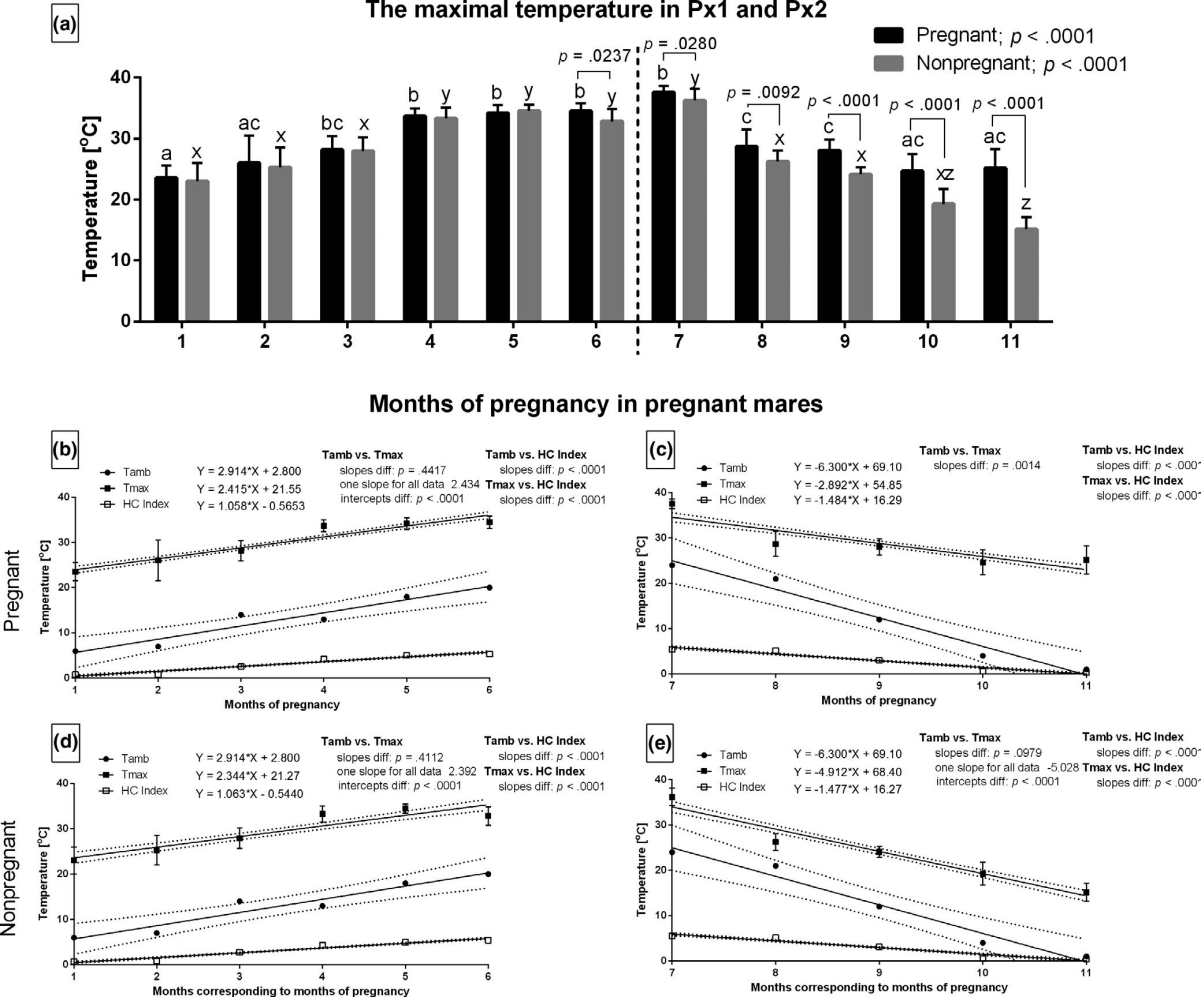
The maximal temperature (Tmax) in Px1 and Px2 in consecutive months of study in pregnant (*n* = 26) and non‐pregnant (*n* = 14) groups. Bars represent mean ± *SD*. Different superscripts within pregnant (a, b, c) and non‐pregnant (x, y, z) groups were statistically different (*p* < .05). Differences between pregnant and non‐pregnant groups were indicated with individual *p* value when *p* < .05 (a). Linear regressions of Tmax, Tamb and HC Index in pregnant (b, c) and non‐pregnant (d, e) groups in the increasing period (b, d) and the decreasing period (C, E)

During the increasing period, the months with the highest Tamb coincided with the highest Tmax in both pregnant and non‐pregnant groups. In the decreasing period, a similar association was noted albeit only in the non‐pregnant group. The Tmax values varied significantly between the two groups of mares from the 6th to the 11th month (Figure 3a). During the increasing period, the linear regression slopes for Tamb and Tmax had similar significance levels (Figure 3b and 3d). The slope for each data set was calculated as 2.434 for the pregnant group and 2.392 for the non‐pregnant group. During the decreasing period, only the slopes of the non‐pregnant group were significantly no different for Tamb and Tmax (Figure 3e), and one slope was calculated as −5.028. The intercepts in the linear regression equation were significantly different (*p* < .001) for those data pairs. Other slopes, especially data pairs with HC Index, varied significantly (*p* < .001).

**FIGURE 4:**
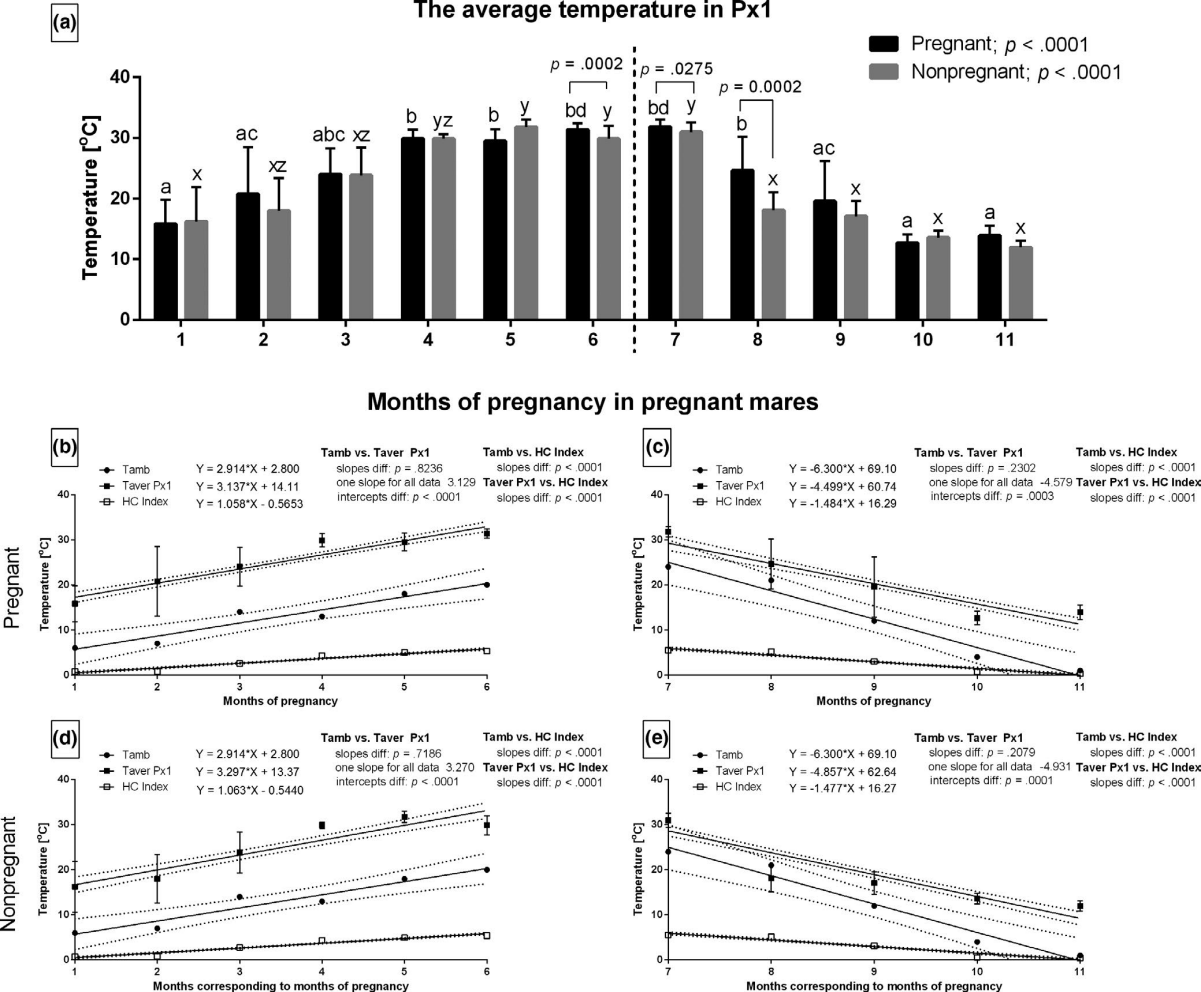
The average temperature (Taver) in Px1 in consecutive months of study in pregnant (*n* = 26) and non‐pregnant (*n* = 14) groups. Bars represent mean ± *SD*. Different superscripts within pregnant (a, b, c) and non‐pregnant (x, y, z) groups were statistically different (*p* < .05). Differences between pregnant and non‐pregnant groups were indicated with individual *p* value when *p* < .05 (a). Linear regressions of Taver Px1, Tamb and HC Index in pregnant (b, c) and non‐pregnant (d, e) groups in the increasing period (b, d) and the decreasing period (c, e)

The months with the highest Tamb values corresponded with the highest Taver Px1 values in both pregnant and non‐pregnant groups. They also fluctuated with the increasing and decreasing periods. The values of Taver in Px1 varied significantly between pregnant and non‐pregnant mares from the 6th to 8th month of study (Figure 4a). The slopes in the linear regression equation for Tamb and Taver in Px1 in pregnant (Figure 4b and 4c) and non‐pregnant (Figure 4d and 4e) groups did not differ significantly. One slope for each data set was calculated as 3.129 and −4.579 for pregnant mares. It was 3.270 and −4.931 for non‐pregnant mares. The intercepts were significantly different (*p* < .001) for all the above pairs of data. Other slopes, especially data pairs with the HC Index, differed significantly (*p* < .001).

**FIGURE 5:**
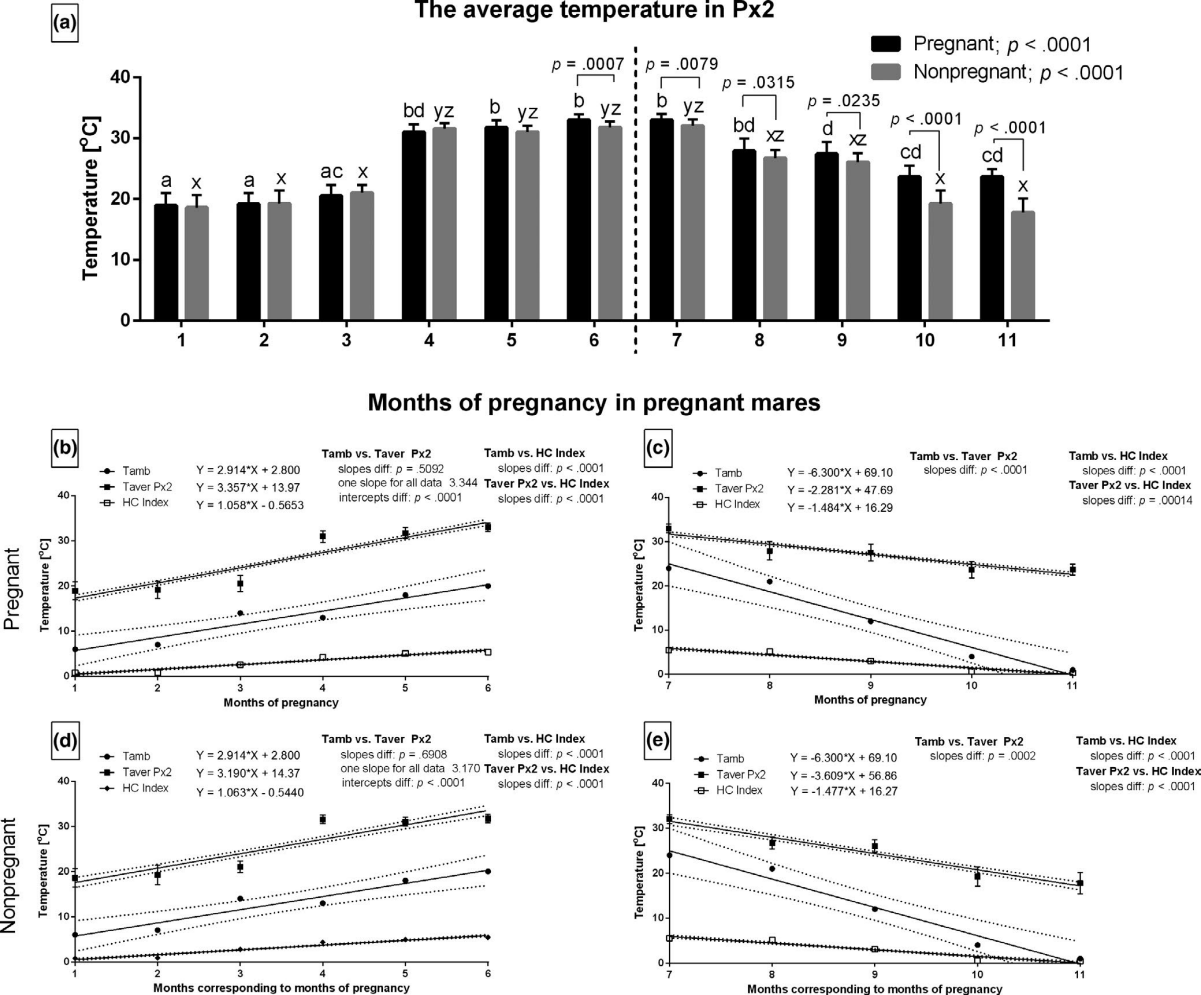
The average temperature (Taver) in Px2 in consecutive months of study in pregnant (*n* = 26) and non‐pregnant (*n* = 14) groups. Bars represent mean ± *SD*. Different superscripts within pregnant (a, b, c) and non‐pregnant (x, y, z) groups were statistically different (*p* < .05). Differences between pregnant and non‐pregnant groups were indicated with individual *p* value when *p* < .05 (a). Linear regressions of Taver Px2, Tamb and HC Index in pregnant (b, c) and non‐pregnant (d, e) groups in the increasing period (b, d) and the decreasing period (c, e)

During the increasing period, the months with the highest Tamb values corresponded with the highest Taver values in Px2 in both pregnant and non‐pregnant groups. This association was not observed during the decreasing period. The values of Taver in Px2 were significantly different between the two distinct mare groups from the 6th to 11th months of study (Figure 5a). During the increasing period, the slopes did not differ significantly for Tamb and Taver values in Px2 between the pregnant (Figure 5b) and non‐pregnant (Figure 5d) group. For those data pairs, one slope was calculated as 3.344 and 3.170, respectively, and the intercepts were significantly different (*p* < .001). Other slopes were significantly different (*p* < .001).

**FIGURE 6:**
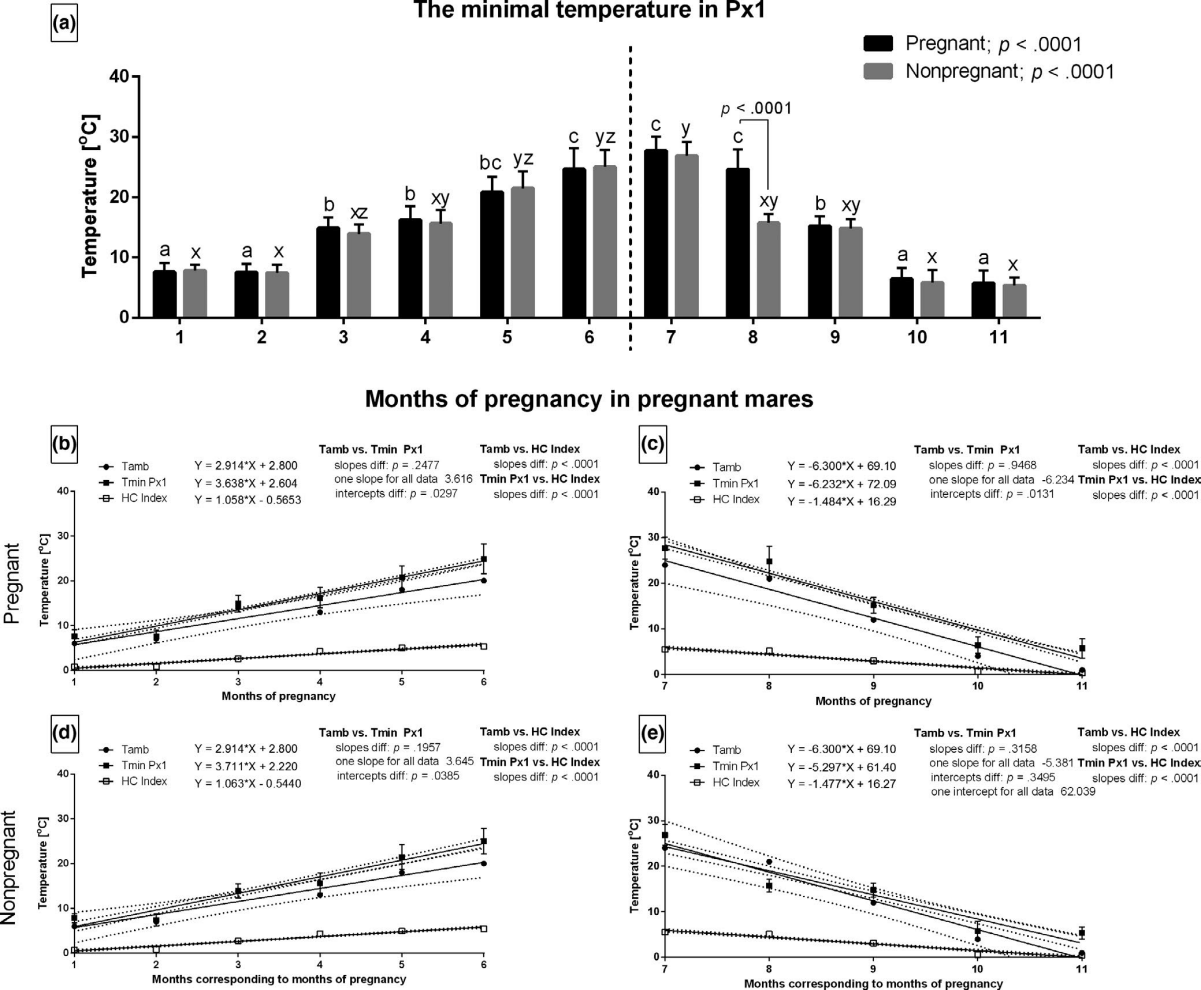
The minimal temperature (Tmin) in Px1 in consecutive months of study in pregnant (*n* = 26) and non‐pregnant (*n* = 14) groups. Bars represent mean ± *SD*. Different superscripts within pregnant (a, b, c) and non‐pregnant (x, y, z) groups were statistically different (*p* < .05). Differences between pregnant and non‐pregnant groups were indicated with individual *p* value when *p* < .05 (a). Linear regressions of Tmin Px1, Tamb and HC Index in pregnant (b, c) and non‐pregnant (d, e) groups in the increasing period (b, d) and the decreasing period (c, e)

The months with the highest Tamb corresponded with the highest Tmin in Px1 in both groups and during both periods. The values of Tmin in Px1 differed only during the 8th month of study between the two groups of mares (Figure 6a). The slopes did not differ for Tamb and Tmin in Px1 between the pregnant (Figure 6b and 6c) and non‐pregnant (Figure 6d and 6e) groups. One slope was calculated as 3.616 and −6.234 for the pregnant group and 3.645 and −5.381 for the non‐pregnant mares. The intercepts did not differ. The only exception was for the Tamb and Tmin in Px1 in the non‐pregnant group during the decreasing period, where one intercept for this data pair was calculated as 62.039 (Figure 6e). Other slopes with the HC Index were significantly different (*p* < .001).

**FIGURE 7:**
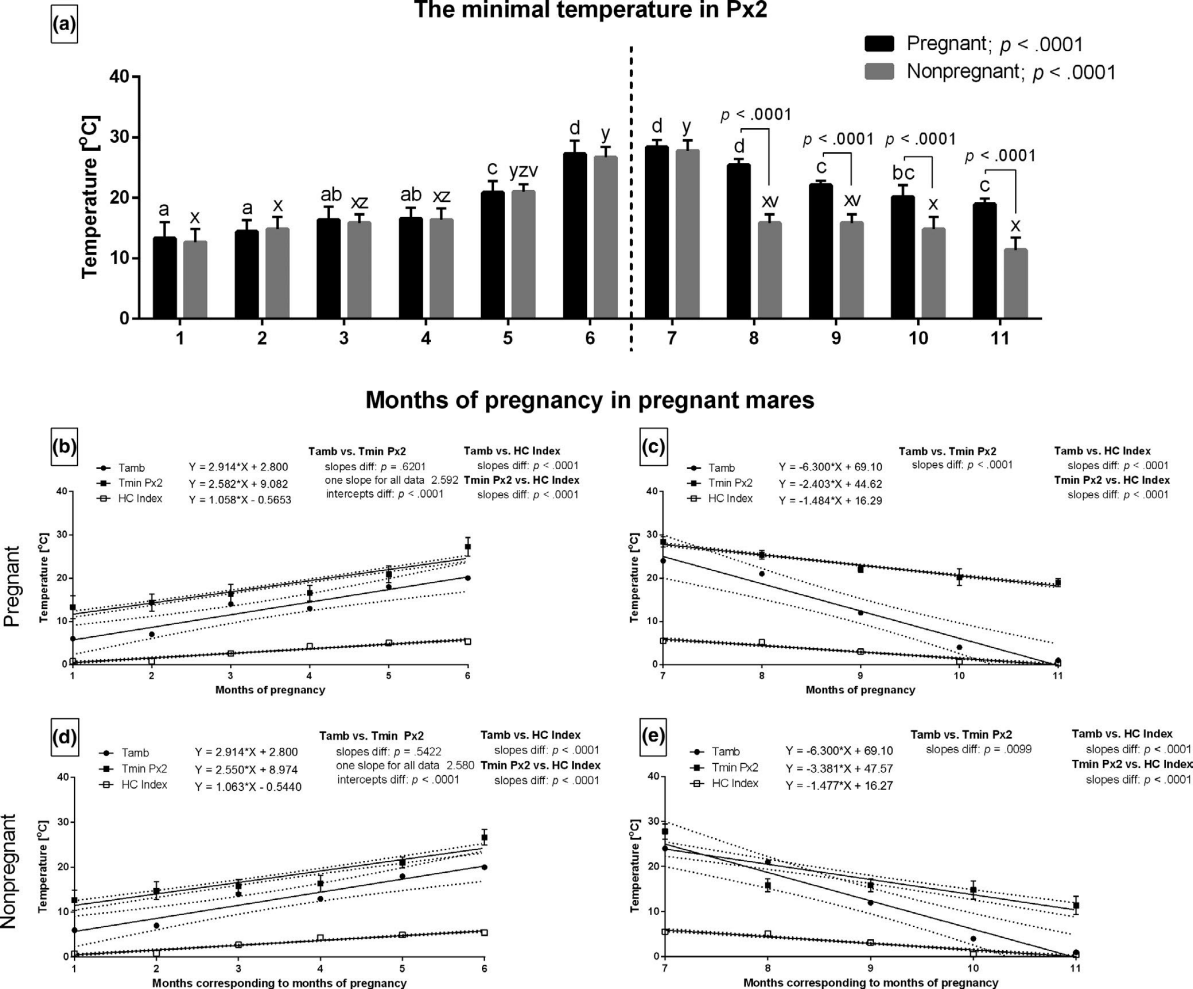
The minimal temperature (Tmin) in Px2 in consecutive months of study in pregnant (*n* = 26) and non‐pregnant (*n* = 14) groups. Bars represent mean ± *SD*. Different superscripts within pregnant (a, b, c) and non‐pregnant (x, y, z) groups were statistically different (*p* < .05). Differences between pregnant and non‐pregnant groups were indicated with individual *p* value when *p* < .05 (A). Linear regressions of Tmin Px2, Tamb and HC Index in pregnant (B, C) and non‐pregnant (D, E) groups in the increasing period (B, D) and the decreasing period (C, E)

During the increasing period only, the months with the highest Tamb corresponded with the highest Tmin in Px2 in both groups of mares. The values of Tmin in Px2 varied significantly between the groups from the 8th to the 11th months (Figure 7a). During the increasing period, the slopes did not differ for Tamb and Tmin in Px2 between the pregnant (Figure 7b) and non‐pregnant (Figure 7d) groups with one slope calculated as 2.592 and 2.580 respectively. The intercepts for those data pairs were significantly different (*p* < .001). Other slopes were also significantly different (*p* < .001).

For the thermographic pregnancy diagnosis using Tmax in Px2, temperature threshold with the highest Se and Sp were determined at the level of 34.0℃ in 6th month (Se 0.68; Sp 0.64), 34.5℃ in 7th month (Se 0.68; Sp 0.64), 27.0℃ in 8th month (Se 0.72; Sp 0.64), 27.0℃ in 9th month (Se 0.76; Sp 0.64), 24.0℃ in 10th month (Se 0.72; Sp 0.73) and 23.5℃ in 11th month (Se 0.76; Sp 0.73) (Table 1). The thermographic pregnancy diagnosis using Taver in Px2 was characterized by higher Se and Sp for the period from 6 to 11 months. The temperature threshold were set at the level of 32.0℃ in 6th month (Se 0.89; Sp 0.62), 32.0℃ in 7th month (Se 0.86; Sp 0.64), 26.5℃ in 8th month (Se 0.80; Sp 0.46), 26.0℃ in 9th month (Se 0.75; Sp 0.46), 22.0℃ in 10th month (Se 0.86; Sp 0.92) and 20.0℃ in 11th month (Se 1.00; Sp 0.92) (Table 2). Finally, Tmin in Px2 allowed to distinguish the pregnant and non‐pregnant group with the highest Se and Sp from 8 to 11 months. The temperature threshold were determined at the level of 17.5℃ in 8th month (Se 1.00; Sp 0.91), 17.5℃ in 9th month (Se 1.00; Sp 0.91), 17.5℃ in 10th month (Se 0.89; Sp 0.91) and 15.0℃ in 11th month (Se 1.00; Sp 1.00) (Table 3).

**TABLE 1:** Temperature threshold used to estimate the sensitivity (Se), specificity (Sp), positive predictive value (PPV) and negative predictive value (NPV) of thermographic pregnancy diagnosis using the maximal temperature (Tmax) in the flank area of the lateral surface of the mares' abdomen (Px2). Temperature threshold with the highest Se and Sp simultaneously marked in bold

Month	Temperature threshold					
6th	33.0℃	33.5℃	34.0℃	34.5℃	35.0℃	35.5℃
Se	0.84	0.72	**0.68**	0.56	0.40	0.20
Sp	0.45	0.55	**0.64**	0.73	0.82	1.00
PPV	0.78	0.78	**0.81**	0.82	0.83	1.00
NPV	0.56	0.46	**0.47**	0.42	0.38	0.35
7th	33.5℃	34.0℃	34.5℃	35.0℃	35.5℃	36.0℃
Se	0.84	0.68	**0.68**	0.52	0.28	0.12
Sp	0.55	0.55	**0.64**	0.82	0.91	1.00
PPV	0.81	0.77	**0.81**	0.87	0.88	1.00
NPV	0.60	0.43	**0.47**	0.43	0.36	0.33
8th	25.0℃	25.5℃	26.0℃	26.5℃	27.0℃	27.5℃
Se	0.88	0.88	0.84	0.80	**0.72**	0.68
Sp	0.27	0.36	0.45	0.55	**0.64**	1.00
PPV	0.73	0.76	0.78	0.80	**0.82**	1.00
NPV	0.50	0.57	0.56	0.55	**0.50**	0.58
9th	26.0℃	26.5℃	27.0℃	27.5℃	28.0℃	28.5℃
Se	0.84	0.80	**0.76**	0.64	0.52	0.40
Sp	0.36	0.55	**0.64**	0.73	0.91	1.00
PPV	0.75	0.80	**0.83**	0.84	0.93	1.00
NPV	0.50	0.55	**0.54**	0.47	0.45	0.42
10th	23.5℃	24.0℃	24.5℃	25.0℃	25.5℃	26.0℃
Se	0.80	**0.72**	0.60	0.48	0.40	0.36
Sp	0.64	**0.73**	0.73	0.82	0.82	1.00
PPV	0.83	**0.86**	0.83	0.86	0.83	1.00
NPV	0.58	**0.53**	0.44	0.41	0.38	0.41
11th	22.5℃	23.0℃	23.5℃	24.0℃	24.5℃	25.0℃
Se	0.84	0.80	**0.76**	0.64	0.56	0.44
Sp	0.55	0.64	**0.73**	0.73	0.82	1.00
PPV	0.81	0.83	**0.86**	0.84	0.88	1.00
NPV	0.60	0.58	**0.57**	0.47	0.45	0.44

**TABLE 2:** Temperature threshold used to estimate the sensitivity (Se), specificity (Sp), positive predictive value (PPV) and negative predictive value (NPV) of thermographic pregnancy diagnosis using the average temperature (Taver) in the flank area of the lateral surface of the mares' abdomen (Px2). Temperature threshold with the highest Se and Sp simultaneously marked in bold

Month	Temperature threshold					
6th	31.5℃	32.0℃	32.5℃	33.0℃	33.5℃	34.0℃
Se	0.94	**0.89**	0.74	0.43	0.29	0.09
Sp	0.46	**0.62**	0.85	0.92	0.92	1.00
PPV	0.83	**0.86**	0.93	0.94	0.91	1.00
NPV	0.75	**0.67**	0.55	0.38	0.32	0.29
7th	31.5℃	32.0℃	32.5℃	33.0℃	33.5℃	34.0℃
Se	0.92	**0.86**	0.64	0.47	0.33	0.17
Sp	0.36	**0.64**	0.64	0.86	0.93	1.00
PPV	0.79	**0.86**	0.82	0.89	0.92	1.00
NPV	0.63	**0.64**	0.41	0.39	0.35	0.32
8th	26.0℃	26.5℃	27.0℃	27.5℃	28.0℃	28.5℃
Se	0.80	**0.80**	0.63	0.57	0.51	0.43
Sp	0.31	**0.46**	0.62	0.77	0.85	1.00
PPV	0.76	**0.80**	0.81	0.87	0.90	1.00
NPV	0.36	**0.46**	0.38	0.40	0.39	0.39
9th	25.0℃	25.5℃	26.0℃	26.5℃	27.0℃	27.5℃
Se	0.86	0.78	**0.75**	0.69	0.69	0.47
Sp	0.08	0.31	**0.46**	0.62	0.69	1.00
PPV	0.72	0.76	**0.79**	0.83	0.86	1.00
NPV	0.17	0.33	**0.40**	0.42	0.45	0.41
10th	22.0℃	22.5℃	23.0℃	23.5℃	24.0℃	24.5℃
Se	**0.86**	0.71	0.60	0.51	0.40	0.31
Sp	**0.92**	0.92	0.92	0.92	0.92	1.00
PPV	**0.97**	0.96	0.95	0.95	0.93	1.00
NPV	**0.71**	0.55	0.46	0.41	0.36	0.35
11th	19.5℃	20.0℃	20.5℃	21.0℃	21.5℃	22.0℃
Se	1.00	**1.00**	1.00	1.00	0.94	0.92
Sp	0.69	**0.92**	0.92	0.92	0.92	1.00
PPV	0.90	**0.97**	0.97	0.97	0.97	1.00
NPV	1.00	**1.00**	1.00	1.00	0.86	0.81

**TABLE 3:** Temperature threshold used to estimate the sensitivity (Se), specificity (Sp), positive predictive value (PPV) and negative predictive value (NPV) of thermographic pregnancy diagnosis using the average temperature (Taver) in the flank area of the lateral surface of the mares' abdomen (Px2). Temperature threshold with the highest Se and Sp simultaneously marked in bold

Month	Temperature threshold					
6th	26.5℃	27.0℃	27.5℃	28.0℃	28.5℃	29.0℃
Se	0.70	**0.58**	0.48	0.39	0.27	0.18
Sp	0.40	**0.50**	0.50	0.70	0.80	1.00
PPV	0.79	**0.79**	0.76	0.81	0.82	1.00
NPV	0.29	**0.26**	0.23	0.26	0.25	0.27
7th	27.0℃	27.5℃	28.0℃	28.5℃	29.0℃	29.5℃
Se	0.81	0.64	**0.56**	0.47	0.28	0.08
Sp	0.36	0.36	**0.45**	0.55	0.55	1.00
PPV	0.81	0.77	**0.77**	0.77	0.67	1.00
NPV	0.36	0.24	**0.24**	0.24	0.19	0.25
8th	15.5℃	16.0℃	16.5℃	17.0℃	17.5℃	18.0℃
Se	1.00	1.00	1.00	1.00	**1.00**	1.00
Sp	0.36	0.36	0.64	0.82	**0.91**	1.00
PPV	0.82	0.82	0.89	0.94	**0.97**	1.00
NPV	1.00	1.00	1.00	1.00	**1.00**	1.00
9th	15.5℃	16.0℃	16.5℃	17.0℃	17.5℃	18.0℃
Se	1.00	1.00	1.00	1.00	**1.00**	1.00
Sp	0.36	0.36	0.64	0.82	**0.91**	1.00
PPV	0.83	0.83	0.90	0.95	**0.97**	1.00
NPV	1.00	1.00	1.00	1.00	**1.00**	1.00
10th	17.5℃	18.0℃	18.5℃	19.0℃	19.5℃	20.0℃
Se	**0.89**	0.86	0.81	0.75	0.67	0.64
Sp	**0.91**	0.91	0.91	0.91	0.91	1.00
PPV	**0.97**	0.97	0.97	0.96	0.96	1.00
NPV	**0.71**	0.67	0.59	0.53	0.45	0.46
11th	12.5℃	13.0℃	13.5℃	14.0℃	14.5℃	15.0℃
Se	1.00	1.00	1.00	1.00	1.00	**1.00**
Sp	0.64	0.82	0.82	0.91	0.91	**1.00**
PPV	0.89	0.94	0.94	0.97	0.97	**1.00**
NPV	1.00	1.00	1.00	1.00	1.00	**1.00**

## 4 DISCUSSION

Bowers et al. (2009) show that the thermographic imaging was able to detect pregnancy in the horse during late gestation, from the 9th month of pregnancy. In this period, Bowers et al. (2009) results were largely confirmative by our findings, which indicated the usefulness of thermography in assessing the body surface temperature of pregnant mares. In the study presented here, the part of limitations of Bowers et al. (2009) research was improved. In this research, the imaging period was extended from 3 to 11 months, the size and homogeneity of groups (one breed and hair coat colour) were increased, the number of assessed thermal features and body areas were increased and the thermal properties of hair coat were taken into account. Moreover, the linear regression was introduced as a more powerful approach than the correlations used in Bowers et al. (2009), since in the regression approach the various measurements were all analysed together across the month of pregnancy. In such an improved experimental model, we demonstrated the differences between the pregnant and non‐pregnant groups from the 6th month using Tmax and Taver as well as from the 8th month using Tmin. This preliminary study estimated the sensitivity and specificity of the thermographic pregnancy diagnosis at a high level for Tmin in Px2 from the 8th month. The use of Taver in Px2 allowed to estimate a lower sensitivity and specificity, but earlier, from the 6th month of pregnancy, while Tmax in Px2 was characterized by the lowest specificity of group distinguishing among the examined features. However, more extensive studies on a larger number of mares are needed to verify these initial estimations.

It should be highlighted, the lack of features of the early thermographic diagnosis of pregnant mares is a limitation of the method, since ultrasound allows to diagnose pregnancy already on the 14th–16th day after ovulation (McCue, 2014). Furthermore, in the case of wild horses, the ability to identify a pregnancy based on thermal imaging is limited by the lack of knowledge about mating (Kirkpatrick et al., 1988), the environmental conditions (Kastelic et al., 1996; Soroko et al., 2017) and seasonal changes in thermal properties of the skin and hair coat (Domino et al., 2020; Jørgensen et al., 2020; Mejdell & Bøe, 2005). Therefore, this preliminary study aimed to determine the associations between ambient temperatures, hair coat features and abdominal lateral surface temperatures in mares.

The optimal temperature for thermographic imaging ranges from 16℃ to 24℃ (Satchell et al., 2015). In equids, the late gestation takes place during the winter months, when the ambient temperature in Poland is much lower (<10℃). Bowers et al. (2009) performed thermographic images of pregnant mares when the ambient temperatures were between 4.2℃ and 28.9℃, whereas in this study the ambient temperatures ranged from 1℃ to 24℃. Bowers et al. (2009) observed greater differences between flank temperatures in pregnant and non‐pregnant mares when Tamb was below 19℃. In this study, differences in Tmax between pregnant and non‐pregnant mares were observed when Tamb ranged from 1℃ to 24℃. The association between Tmax and Tamb was only seen in the non‐pregnant group. Although it was possible to distinguish the pregnant from the non‐pregnant mares regardless of the Tamb, its effect should not be ignored and should be considered in such comparisons.

Environmental conditions such as ambient temperatures (Satchell et al., 2015) and insulation drive seasonal fluctuations in hair coat lengths (Jørgensen et al., 2020). These may impact the reliability of thermal images taken in a natural environment, that is, the wildlife equids natural habitat. Jørgensen et al. (2020) reported that breed type influenced hair coat length and weight. They also noted that the body condition score affected the hair coat's quality and suggested that these variables were important in limiting the amount of radiant energy emitted from the body's surface. This study examined one breed of comparable healthy animals; therefore, the relationships described can be attributed to differences in hair length, and not to the innate properties of the hair (Jørgensen et al., 2020). In primitive horse breeds living on reserves, such as the Polish Konik (Pasicka, 2013), the area of the body covered with short hair increased in April and May and decreased in September and October (Stachurska et al., 2015). In other native pony breeds, such as the Icelandic horses, the largest average coat length was noted in December, and the smallest was seen in June (Mejdell & Bøe, 2005). In both studies, there was a significant negative correlation between the hair coat length and the average ambient temperature (Mejdell & Bøe, 2005; Stachurska et al., 2015). In this study, the highest hair coat length values were noted during the 1st, 2nd, 10th and 11th months of the study, which were February, March, November and December respectively. Analogically, the shortest lengths were recorded from June to September, which is consistent with recent research findings. The relationship between the length of the hair coat and Tamb could not be calculated with linear regression during a specific time point due to the inversely proportional relationships that bound these variables (Mejdell & Bøe, 2005; Stachurska et al., 2015). Instead, the HC Index was calculated. The immense variability in slope values between the HC Index and temperatures versus the little variability in slope values between Tamb and surface temperatures revealed less dynamism associated with Tamb for the HC Index than surface temperatures.

This preliminary study found no significant association between the HC Index and surface temperature. An association between Tamb and surface temperature was observed for Taver in Px1 and Tmin in Px1. Among the measured temperatures, Taver in Px2 and Tmin in Px2 seemed less dependent on Tamb. However, there was no evident association noted in both pregnant and non‐pregnant groups. It is worth highlighting that Tmax in Px1 and Px2 correlated with Tamb in non‐pregnant mares, which contrasted with the pregnant mares. This could be potentially explained by an opposite hair coat growth pattern in the flank area (Górecka et al., 2006). This could lead to lower local thermal insulation, which in turn would reduce the radiant energy emission (Jørgensen et al., 2020). Since there were differences in Px2 temperatures between both mares' groups from the 6th or 8th month of study to the end of the study, those thermal features should be considered in further research. Research involving comparisons of thermal features of the brushed and non‐brushed lateral surfaces of the abdomen (Soroko & Howell, 2018) and comparisons of indoor and outdoor thermographic images (Tunley & Henson, 2004) will be required to gain a better understanding of the relationship between imaging conditions and thermal features of pregnant mares in the natural environment.

## 5 CONCLUSIONS

The associations between ambient temperature and lateral surface temperatures of the mares' abdomen were more pronounced when the whole area of the abdomen (Px1) was considered than when only the flank area of the abdomen (Px2) was measured. The flank area appears to be more suitable for thermal imaging in pregnant mares due to the seasonal fluctuations in hair coat lengths. There was no evidence of parallel changes in hair coat features and measured temperatures. Moreover, the Tmin in the flank area seems to be a sensitive feature that distinguishes between pregnant and non‐pregnant mares only from the 8th month of pregnancy. The Taver in the flank area was characterized by lower sensitivity but the earlier distinguishes, from the 6th month of pregnancy. Therefore, the measured surface temperatures, Taver and Tmin in Px2 deserve attention in further research on thermographic imaging of pregnant mares in the natural environment.

## CONFLICT OF INTEREST

None of the authors has any conflict of interest to declare.

## AUTHOR CONTRIBUTIONS

The idea for the paper was conceived by MM and MD. The experimental protocol was designed by all the authors. The data were obtained by MM and OWP. It was statistically analysed by MD and TJ and discussed by all authors. The paper was written by MM and MD, and critically revised by all authors.

## ETHICAL APPROVAL

All were performed in accordance with the protocol approved by the II Local Ethical Committee on Animal Testing in Warsaw (Permit Number: WAW2/007/2020 from 15.01.2020) on behalf of the National Ethical Committees on Animal Testing.

## Data Availability

The data that support the findings of this study are available from the corresponding author upon reasonable request.
